# The willingness and barriers to collaborate in the care of frail older adults: perspectives of primary care professionals

**DOI:** 10.1186/s12877-023-04163-y

**Published:** 2023-08-11

**Authors:** Leen De Coninck, Anja Declercq, Leen Bouckaert, Carola Döpp, Maud J.L. Graff, Bert Aertgeerts

**Affiliations:** 1 Department of Public Health and Primary Care, KU Leuven, Kapucijnenvoer 7, Leuven, 3000 Belgium; 2 CEBAM Belgian Center for Evidence-based Medicine vzw, Kapucijnenvoer 7, Leuven, 3000 Belgium; 3 Department of Occupational Therapy, Artevelde University of Applied Sciences, Voetweg 66, Ghent, 9000 Belgium; 4 LUCAS Center for Care Research and Consultancy & CESO Center for Sociological Research, KU Leuven, Minderbroedersstraat 8, Leuven, 5310 Belgium; 5 Scientific Institute for Quality of Health Care, Department of Rehabilitation, Radboudumc Research Institute, Radboud University Medical Center, Houtlaan 4, Nijmegen, 6525 XZ the Netherlands

**Keywords:** Primary care, Inter-professional collaboration, Frailty, Barriers

## Abstract

**Background:**

This study investigated the perspectives of primary care professionals, in particular general practitioners, registered nurses, physiotherapists and occupational therapists, on inter-professional collaboration, the barriers and the facilitators they perceive in the care of the frail older population.

**Methods:**

We conducted a qualitative study. In-depth interviews with healthcare professionals were performed, using open-ended questions about their perceptions on the care of frail older adults and inter-professional collaboration. Data was analyzed following the Basic Logical Model of Abduction and Creswell’s coding method.

**Results:**

Healthcare professionals indicated that when they explored problems complementary to the reasons for older people to contact a healthcare professional, these additional problems often seemed to be the main problem. They also stated that there was too little inter-professional collaboration in the care of complex chronic issues and lack of a shared vision on collaboration. Collaboration is still limited too much to contacting established professions. Health information technology can support both, inter-professional collaboration and working on an evidence-based manner. It can also be a facilitator to inform patients. The availability and use of health information technology differs between the professions. Success factors and barriers for sustainable collaboration were identified on several levels, namely innovation, individual, professional, patient, social context, context of the organization, economic and political context.

**Conclusions:**

Our study shed light on the willingness and barriers in collaboration of healthcare professionals in primary care for older adults. There is little inter-professional collaboration, despite the willingness of the healthcare professionals to collaborate.

**Supplementary Information:**

The online version contains supplementary material available at 10.1186/s12877-023-04163-y.

## Background

Healthcare systems across the world, among which Belgium, are challenged by an ageing population. Studies show that complex chronic issues require an inter-professional collaborative or an integrated approach to caregiving [1–3]. Integrated care holds the potential to enhance the quality, safety, and efficiency of healthcare delivery in complex chronic issues while improving patient experience and outcomes. WHO defines integrated people-centered health services as “providing health services at the right time, in the right place in the right way, locating services close to people and communities”. Integrated care encompasses a comprehensive perspective on health, transcending the mere focus on diseases or interventions, to recognize the entirety of an individual and the ongoing care required across their entire lifespan [4].

Moreover, recent studies on interprofessional collaboration in elderly primary care indicate that enablement models of integration can be successful in activating positive change towards independence for the older adult with complex needs [5]. Even though prevention of safety risks is not an explicit goal of the integrated care program studies show that integrated care address risks in social and environmental domains [6]. Older adults desire accessible, efficient and coordinated care that caters to their needs and preferences, while keeping in mind their rights and safety [7]. So, integrated care programs support the wish of older people: to live safely at home.

Interprofessional digital communication tools offer opportunities to support interprofessional collaboration. Health professionals indicate to be better informed about patients’ current situations when using digital communication tools. However, the number of digital systems professionals simultaneously use, and different work agreements hamper tool use [8, 9]. Health professionals often have their own discipline-specific tools with varying functionalities. This lack of interoperability of systems is a major barrier and might result in old-fashioned forms of communication such as using the phone [10].

It is a key challenge in general for both policy makers and healthcare professionals to create an optimized professional setting for pursuing high quality of complex primary care. For instance, policy can have a significant role in realizing the tools’ long-term use [9]. The Chronic Care Model (CCM) describes the components needed for qualitative complex chronic care and to improve patient’s outcomes. The core components of the CCM include both community resources and policies, and health system organization namely self-management support, a delivery system design, decision support and a clinical information system. The components of CCM are essential in fostering productive interactions between healthcare professionals and their patients. This includes informed and empowered patients and a prepared pro-active practice team. The CCM states that interventions that encourage people to acquire self-management skills are essential in chronic illness care [11–14].

European healthcare professionals and care-infrastructure are not sufficiently prepared to qualitatively treat the older population with chronic conditions [15–17]. The way primary care is organized and financed at this moment hinders the accessibility of certain healthcare professions which are proven to be effective in elderly care [18]. Healthcare professions that encourage people to acquire self-management skills are not adequately included in integrated care teams although enablement models of integration shows to be successful in activating positive change towards independence for the frail older adult [5]. Occupational therapists are healthcare professionals whose main goal is to enable engagement in meaningful daily activities within the individuals’ living environment, thereby fostering their health and well-being [19]. It has been shown that inter-professional interventions which involve occupational therapy in primary care are effective in delaying institutionalization of frail older adults [20]. Primary care occupational therapy can prolong the time older adults remain at home for at least 6 months [21]. Despite the evidence, occupational therapy in primary care remains an underused profession in most European countries, among which Belgium [18]. Consequently, there is a need for restructuring primary care to provide all relevant health professionals the opportunity to deliver optimal inter-professional care for frail elderly.

Beside the underuse of certain healthcare professionals, there are more barriers to implement an integrated chronic care model in primary care in Belgium. Defined barriers are patient empowerment and health promotion, inter-professional collaboration, integration and continuity between hospital and primary care and integration and continuity between healthcare and social welfare [17]. In the context of patient empowerment, a qualitative study on the perspectives of older adults on functioning, social participation and health provided data of interest for other involved healthcare providers to increase adherence of the target population [22].

The barriers on inter-professional collaboration can be defined as challenges of definition and awareness of one another’s roles and competences, sufficient communication and shared information, confidentiality and responsibility, team building and inter-professional training, long-term funding and regulations, and joint monitoring [23, 24]. In less-involved healthcare professions, such as occupational therapy, there is an additional challenge in terms of awareness of each other’s roles and competences, as knowledge of one another’s roles is considered a prerequisite for trustworthiness [23].

Following Grol’s (2004) barriers to and incentives for change, various components need to be addressed when planning changes in practice, namely the characteristics of the patients and the professionals involved, the level of innovation, the social context, the organizational context, and the economic and political context. The characteristic of the patient implies their knowledge, skills, attitude and compliance. The characteristics of the individual professionals is about awareness, knowledge, attitude, motivation to change and behavioral routines. The level of innovation concerns what advantages the innovation brings in practice, the feasibility, credibility, accessibility and attractiveness. The social context is about the opinion of colleagues, culture of the network, collaboration and leadership. Organization of care processes, staff, capacities, resources, structures are components that describe the organizational context. The financial arrangements, regulations, policies describes the economic and political context [25].

There is still a need for insight in other barriers of changes in practice, among which the willingness of healthcare professionals to collaborate. The characteristics of the individual professional and the social context are substantial. However, the willingness to collaborate can also be influenced by the other components as defined by Grol. Therefore, this study aims to understand the perspectives of primary care professionals of inter-professional collaboration in care for the frail older population and considers both the well-known and less-involved primary care professionals relevant for the frail older population.

## Objective

This study investigated the perspectives of primary care professionals, in particular general practitioners, registered nurses, physiotherapists, and occupational therapists, on inter-professional collaboration, and the barriers and facilitators they perceived in the care of the frail older population.

## Methods

### Design

In-depth interviews with healthcare professionals were performed, using open-ended questions about their perceptions on the care of frail older adults and inter-professional collaboration.

The theoretical framework underpinning this study is the Basic Logical Model of Abduction which uses abductive analysis as its qualitative data analysis approach [26]. Abductive analysis aims at generating novel theoretical insights through a dialog between sensitivity for cultivated theory and methodological heuristics [27]. In this way, the Basic Logical Model of Abduction emphasizes that research can be both inductive and deductive. Abductive analysis allows researchers to let themes, patterns and categories emerge from data (inductive) and, on the other hand, rely on existing analytical categories obtained from previous theories (deductive). Insightfully abducting during analysis consists of developing themes, codes and categories that structure data. When organizing and structuring the analyzed information, it might turn out that it fits within existing models. These existing models supply insights which, in turn, lead to further analysis, so that broader and more extensive knowledge is gained. Abduction challenges researchers to develop their theoretical repertoires throughout the research process rather than setting all preconceived theoretical ideas aside during the research project [28, 29].

Our investigation is influenced by two theories, the CCM of Wagner (2001) and Grol’s potential barriers to and incentives for change when planning a complex intervention. The data obtained from the interviews can be linked to the essential components of high-quality care to improve productive interactions as defined in the CCM by Wagner. Effective communication and collaboration are essential for a prepared and proactive practice team to pursue sustainability and achieve informed and empowered patients [11, 12]. The codes identified during analysis encompassed communication and collaboration between patient and professional, and among professionals. The data can also be linked to the obstacles and motivations for change across various healthcare levels, as defined by Grol [25]. The codes identified during the analysis encompassed the individual healthcare professional, the patient, the innovation itself, the social context, the organizational context, and the economic and political context.

### Sample selection and participants

The respondents were included if they met the following criteria: being a general practitioner, registered nurse, physiotherapist, or occupational therapist; being employed in primary care for at least three years; having experience with older adults with chronic health issues.

Purposive sampling was used, as we opted to strive for the greatest possible diversity and not for saturation. The network of the researchers and primary care professionals was addressed for recruitment. We set a minimum of twelve interviews, spread equally across the four professions. In the selection determination, we considered equal distribution of gender, type of practice (individual, mono-disciplinary or multidisciplinary practice), type of employment (self-employed or employee), and location of the practice (city, urban or rural). No two employees from the same workplace were included.

Respondents were informed by phone about the project and about what would be expected of them. If they agreed to participate an appointment was made for a face-to-face interview. Written informed consent was obtained prior to the interview. Thirteen respondents were contacted. One respondent refused to participate. This person did not give a reason. The interview was performed by an experienced researcher in the respondents’ workplace.

### Data collection

An interview guide was developed to elicit the participant’s experiences, thoughts, and perceptions. (Supplement 1) The interviews lasted 40 min on average, were audiotaped and transcribed verbatim.

The in-depth interview guide questions were based on literature about facilitators and barriers influencing collaboration, as well as on the outcome of our own investigations on the perspectives of older adults on functioning, social participation, and health [22, 30–32].

### Data analysis

The researcher performing the interviews also transcribed the audiotapes. The interviews were then analyzed without using software. Data were analyzed following Creswell’s methodology (2013) in which the steps of the analysis process are data management, reading and taking notes, describing of themes, classifying and interpreting, reporting and visualizing [33].

Two researchers read and reread the transcripts to gain a comprehensive understanding of the content and context of the data. Subsequently, they selected the transcript of the interview of two respondents with divergent profiles. The researchers independently analyzed the text, made notes (short sentences, ideas, or core concepts) and attributed initial codes to the two interviews. The findings were discussed in detail to reach agreement on how to continue analyzing the interviews. The researchers divided the data into themes and text was grouped under meaningful categories. One researcher then analyzed the remaining ten interviews based on the agreement that emerged from the discussion. During the analysis process, subthemes were derived from the data using abductive reasoning. The second researcher reviewed this analysis and discussed alternatives with the first researcher. To ensure the reliability and validity of our analysis the findings are compared with existing theories to validate our interpretations. So, we gradually discovered theoretical frameworks to operationalize concepts of the CCM of Wagner (2001) and the potential barriers at various levels when planning an evidence-based complex intervention of Grol (2004). (Supplement 2) In the final phase of the cycle, the essence was captured in summarizing and presenting our findings in a coherent manner [14, 25].

### Reflection on the role of the researchers

The research group consists of a multidisciplinary team, knowledgeable in the fields of sociology of health, occupational therapy, gerontology, and family medicine. As a result, data analysis was enriched by various academic and professional backgrounds.

### Ethics approval and consent to participate

The study was approved by the ethical committee of the University Hospitals of Leuven, Belgium (S58057). Written and verbal information about the research and an opportunity for questions were given before informed consent was obtained by participants. Anonymity was assured by removing all participant information from the transcripts that could lead to identification.

The lead author (the manuscript’s guarantor) affirms that the manuscript is an honest, accurate, and transparent account of the study being reported; that no important aspects of the study have been omitted; and that any deviations from the study as it was designed (and, if relevant, registered) have been explained.

## Results

### Demographic characteristics

Twelve participants are selected. See Table 1 for the characteristics of the participants included in the study.

**Table 1:** Characteristics of the respondents (n = 12)

Participant	Gender	Profession	Type of practice	Type of employment	Location
1	Male	General practitioner	Gen. practitioner group practice	Self-employed	Rural area
2	Male	General practitioner	Multi-disciplinary practice	Self-employed	Suburb
3	Female	General practitioner	Multi-disciplinary practice	Self-employed	Suburb
4	Female	Registered nurse	Registered nurse group practice	Employee	City
5	Female	Registered nurse	Multi-disciplinary practice	Employee	City
6	male	Registered nurse	Registered nurse group practice	Self-employed	Suburb
7	Female	Occupational therapist	Multi-disciplinary practice	Employee	City
8	Female	Occupational therapist	Multi-disciplinary practice	Employee	City
9	Female	Occupational therapist	Multi-disciplinary practice	Employee	Suburb
10	male	Physiotherapist	Physiotherapy group practice	Self-employed	Rural area
11	Female	Physiotherapist	Physiotherapy group practice	Self-employed	Suburb
12	Female	Physiotherapist	Physiotherapy solo practice	Self-employed	Suburb

### Reasons for older people to contact a healthcare professional

Although medical and functional problems are primary reasons for visiting a general practitioner, cognitive, mental, and social problems are also reported. Medical reasons are commonly cardiac or orthopaedic problems. Functional reasons are fall problems, balance problems and walking disabilities, and not being able to live independently anymore. Cognitive and memory problems, dementia, and fear of falling are mental problems older people indicate. Social reasons are mainly no longer being able to leave the house, less social participation, and loneliness. The burden of the informal caregiver, often the partner, is another reason to contact a general practitioner. During the consultation the general practitioner sometimes detects underlying issues such as reduced cognition.

The initial contact with the physiotherapist usually goes via referral by a general practitioner. Fall problems and mobility are the main contact reasons for physiotherapy, and further cardiac problems, orthopaedic problems, and being isolated.

Nurses are in most cases contacted by the informal carer on referral of the general practitioner. Nurses say that the typical nursing actions such as injections and wound care are often the initial reason for consulting them. Managing medication, hygiene care and burnout of the informal caregiver are also frequent reasons for being contacted. Loneliness is often an underlying factor for appealing a nurse.

The occupational therapist, who is often contacted via social services, express mainly being consulted when functional, social, mobility and/or cognitive problems arise or when the burden on the informal caregiver becomes too heavy. The medical reason is often complex chronical issues, e.g., an older adult with stroke with permanent residual disabilities. Falls problem are often present.

All healthcare professionals report that they usually detect other problems underlying to the initial problem. Healthcare professionals say that when they explore the additional problems, these additional problems often are the main problem.

*‘For a start, the reasons they contact us are often physical problems. We are initially consulted for a wound on the coccyx, but it soon becomes apparent that much more is happening, the person is sedentary, he cannot vouch for his self-care, has few social contacts, is lonely, the burden on the family becomes too heavy, ….*’ (Registered nurse).

### Inter-professional collaboration in general

All healthcare professionals find that there is not enough collaboration in the care of complex chronic issues. They specify that information exchange is essential to adjust the treatment. Information sharing usually limits itself to problems, but it is also important to know what goes well. A general practitioner mentioned a lack of a shared vision on collaboration. Collaboration is still limited too much to contacting only a few professions.

*“Collaborating … insufficient. We are trying to make some progress, but that asks joint vision, I notice that we still have too less vision to collaborate. What we do is contact the nurse and physiotherapist. The professions we were taught during our training.”* (General practitioner).

Additionally, several health professionals demonstrate that, with the aim of closing the gap, local initiatives are taken to get organized. One healthcare professional argued that not only organized meetings are useful. A talk with other healthcare professionals by co-incidence in the workplace can also be fruitful. One healthcare professional communicated that she resigned due to lack of structured communication and collaboration where it concerned patient matters. She felt that she could not provide the desired quality of care in this way and applied for a job in a team where collaboration was highly regarded.

*‘My patients received high-quality physiotherapy treatment, but I noticed that there was more going on and that collaboration with the general practitioner or social worker was necessary. In that practice there was no room for communication with external partners. Now I work in a practice where there is attention for communication and collaboration. I’m convinced this is a precondition in elderly care.’* (Physiotherapist).

Healthcare professionals who work in mono- or multidisciplinary primary care practices regularly participate in internal team meetings on patient related matters. External inter-professional meetings on patient-related matters are sometimes organized upon discharge from the hospital or in complex situations. The care coordination of the health insurance organization organizes these inter-professional meetings. Healthcare professionals from the hospital’s discharge team, the informal caregiver, general practitioner, and registered nurse frequently participate at these inter-professional meetings. Physiotherapists sometimes participate. Occupational therapists are rarely invited to these meetings. One occupational therapist who also has a part-time job as care coordinator of a health insurance organization, signify that, due to task demarcation, she cannot assume her role as occupational therapist in cases where she is asked to act as care-coordinator.

*‘Collaboration, yeah, it’s kind of necessary, but in practice it’s very difficult. On the phone, with caregiver services… You pass on the information, but you seldom get information back. There is no exchange of ideas…. I also have a colleague who is a social worker. We both work at same organization. To work efficiently, we sometimes divide the tasks. She does the start-up and subsequently I take it over.’* (Occupational therapist).

The general practitioners mark that they mostly collaborate with primary care nurses, physiotherapists, and the informal caregivers. When a patient is discharged from hospital, general practitioners often have discharge meetings with the hospital staff. Regularly, general practitioners also collaborate with dieticians and the family care service.

The registered nurses collaborate mostly with the general practitioners and family care services. They express sometimes collaborating with physiotherapists, and seldomly with occupational therapists.

*‘When we notice someone becoming more dependent, we also try to involve family care service, even if that is only for two hours a week, and in this way, we also try to get a larger network among the patients.’* (Registered nurse).

Physiotherapists also indicate that they collaborate with general practitioners. The physiotherapists, as is the case for nursing, need a referral of the general practitioner.

Occupational therapists mostly collaborate with social workers and family care services. They occasionally are in contact with physiotherapists. Occupational therapists indicate that, during their interventions, they receive a lot of information from family care services. These people know the older adult well because they spend more time at the older adult’s house, and they are sometimes there when the occupational therapist performs his intervention.

### Collaboration with lesser-known healthcare professions

Two of the general practitioners, all registered nurses and all physiotherapists indicate that primary care occupational therapy is almost unknown territory for them. One general practitioner has an occupational therapist in their team. This occupational therapist is involved in prevention in general, falls prevention in particular, mobility, and physical posture and ergonomics. Two general practitioners who do not collaborate with an occupational therapist, admit not knowing how to contact them. Also, the nurses and physiotherapists admit not knowing how to contact an occupational therapist.

*‘And I think that they do not know what occupational therapy is, what occupational therapists exactly do in primary care … At home with one of my clients where I was consulted for a secondary table, I met the physio. I introduced myself and the physio told me the general practitioner asked him to look for a wheelchair, but he didn’t know anything about wheelchairs. I told him I’d look for it. You see, there’s a demand, but if you don’t meet accidentally…’* (Occupational therapist).

After being informed on the role of occupational therapy in primary care, all healthcare professionals show their interest in referring to and collaborating with an occupational therapist. They confirm that it is desirable that occupational therapists are known in the region.

Two occupational therapists indicate that they regularly inform general practitioners on the occupational therapy intervention, but never receive any response. Although the occupational therapists state that they are seldom invited to an inter-professional consultation, they do advocate the added value of their presence and once general practitioners know them, they show an interest in collaboration. One occupational therapist tells that at an external multidisciplinary meeting where that person acted as case manager, a general practitioner showed interest in the role of the occupational therapist.

*‘Last time at a patient meeting, I really had the feeling ‘My advice is finally being heard!‘. The general practitioner responded to something I suggested and emphasized it. And the fact that all other disciplines heard it, gave me a sense of recognition. After all, we all work for the benefit of that one patient.* (Occupational therapist)

Occupational therapists communicate that the informal carers, family care service and social workers possess a lot of useful information that may improve the quality of their intervention. Informal caregivers and family care services spend the most time with the older adult and know a lot of that person. The social workers are interesting because they are often the first to map out the complexity of the problem.

### Use of health-information technology

Health information technology can support both, inter-professional collaboration and working on an evidence-based manner. It can also be a facilitator to inform patients.

#### Electronic data registration and sharing

Except for the occupational therapists, all interviewed healthcare professionals use a profession specific labelled electronic patient registration system. The registered nurses of monodisciplinary practices inform that their record is part of the central patient record. The self-employed registered nurse and self-employed physiotherapist declare that they keep data in hard copy as well as electronically. They emphasize that they either don’t have the time to fill in the electronic record correctly or see the electronic registration of data as a barrier. One physiotherapist prints his therapy report to hand over to the general practitioner.

*‘A computer between the older adult and myself creates distance when I talk to him. I use pen and paper to write things down and to explain’* (Physiotherapist).

The healthcare professionals working in a multidisciplinary team indicate that they use a central record where each professional add the relevant information for his discipline. The healthcare professional only has access to that part of the information that is relevant for their intervention.

The occupational therapists register their data into a central patient record system of their place of employment to the degree that is possible. Some occupational therapists individually developed a digital occupational therapy record form. This digital record can either be a structured electronic tool or single Word-template.

Safely sharing electronic data among all healthcare professionals is limited possible in Belgium. The data exchange e.g., occupational therapists and physiotherapists often initially takes place by telephone, by mail or – where applicable – during an inter-professional consultation. Within the multidisciplinary group-practices, certain data is shared through the central electronic patient record. Healthcare professionals admit not being aware of all the possibilities of electronic patient records. Two healthcare professionals who have a professional IT-registration tool indicate that they do not use them consistently. The reason they give is low usability. They feel like distancing themselves from the patient using IT during the consultation.

Aspects that are perceived as useful, are the automatic registration of the number of treatment sessions the patient got, the electronic diary and – in the case of mono- or multi-disciplinary group practices- sharing data.

One healthcare professional declares a refusal to share data electronically out of respect for the patient’s privacy. This healthcare professional believes contacting other healthcare professionals over the phone to be a better alternative.

#### Health-information technology to support quality of care

Except for one person, the term evidence-based practice is known by all interviewed healthcare professionals. The application of electronic devices to implement the evidence-based practice (EBP) principles varies among the various professional groups.

General practitioners use electronic databanks with evidence-based data. Two of the interviewed general practitioners have IT-tools that automatically link the evidence-based data to the electronic health record of the patient (Evidence linker). These healthcare professionals also use an electronic Decision Support System (DSS). The physiotherapists indicate they rely on the information and training that their scientific professional association provides. The registered nurse who works in a structured primary care organization indicates the team members contact the organization’s central office when they require scientifically supported information for an intervention. This nursing organization develops protocols on which the at-home registered nurses base their treatment. The occupational therapists indicate attending training and searching evidence-based information on their own through several databases. They also indicate that finding this information is difficult as their search usually does not provide the expected results.

*“The guidelines within our inter-professional practice are ‘you work evidence-based’ and that’s where it ends. We expect that everyone who works in the practice works evidence-based. With all due respect to what others think about that, I don’t think that what we say is the only truth but what we do is based on that (EB).’* (General practitioner).

### Incentives and barriers for sustainable collaboration

The interviewed healthcare professionals identified success factors and barriers for collaboration, which we listed according to the various levels of healthcare of Grol and colleagues (2004).

#### Innovation

Concerning electronic data sharing, healthcare providers indicate that the electronic record must be accessible both, in the office and at the patient’s home. A smooth use of the device, meaning no errors in the program and easy to use, such as clicking on pre-programmed rubrics, is also a precondition. Pre-programmed rubrics are preferrable provided that all necessary information is covered within these rubrics.

*“An additional problem is the internet connection at the home of the older adult. Hardly any older adult I visit has an internet connection. Only when a younger person lives at the same place, there is internet connection.*“ (Physiotherapist).

All healthcare professionals indicate that it must be possible to decide tailor-made which data to share with who, but this depends always on the condition the patient allows data to be shared. Respecting privacy is the highest priority for all interviewed healthcare professionals.

A threshold that is indicated, is the accessibility of lesser-known professional groups. General practitioners, registered nurses and physiotherapists indicate they are not familiar with contacting occupational therapists. They often do not know local primary care occupational therapists and do not know how to find them. They indicate that if they do not know the way, it will be almost not possible for the older adult to contact an occupational therapist.

*“Honestly, no service has ever suggested working with an occupational therapist … Sometimes I think “occupational therapy will be helpful in this case”, but how should I reach them? I really do not know it. Not knowing a local primary care occupational therapist is a barrier for referring.”* (General practitioner).

An advantage is that the specific value in the context of health and wellbeing of an occupational therapy intervention is confirmed by all interviewed professionals. Another advantage is that an occupational therapist visits the older adult a limited number of times to achieve the stated target. A general practitioner indicates that one or two occupational therapists who operate in one area might increase accessibility and can increase a sustainable collaboration.

#### Individual professional

All healthcare professionals unanimously agree on the importance of collaboration and emphasize the willingness to collaborate when it concerns the older adult with complex chronic issues. In line with this, they argue that sustainable collaboration requires effort from multiple actors in many areas and that the better you know each other, the easier to communicate and collaborate.

The healthcare professionals are aware of their behavioral routines in contacting the best know professions among which general practitioner, registered nurse, and physiotherapist. Dieticians and occupational therapists are less contacted. In addition, it is not clear how to contact an occupational therapist.

Most of the healthcare professionals do not experience many barriers in electronic data sharing, as long as the patient is informed and gives his consent on which data can be shared with whom, and the healthcare professionals can decide to protect data themselves.

*‘Honestly, I do not see downsides to electronic data sharing. Of course, it is the patient who decides what is and what is not shared. The condition is that the patient is informed and gives his consent.’* (General practitioner).

#### Patient

Healthcare professionals indicate that admitting that one becomes help dependent is a barrier for that person to appeal for supplementary healthcare provision.

*“Sometimes I have the feeling that for some older adults, if you do not talk of a problem, the problem does not exist. Even if you are convinced that the person knows the problem. This makes it difficult to refer for supplementary healthcare provision”* (Registered nurse).

Healthcare professionals also indicate that knowing the different professions or being introduced to them by someone they trust is a facilitator for the patient to accept that various health professions are involved.

#### Social context

Two healthcare professionals suggest shadowing another profession for a day to have more insight in, and respect for the other profession.

Several healthcare providers pose that an older adult will accept collaboration with a healthcare provider who is not yet involved more easily, when a trusted healthcare provider or a peer informs them that a certain treatment will be an added value for them.

One general practitioner who works in an inter-professional healthcare team stipulates that working in a team improves job satisfaction. The fact that healthcare providers are surrounded by people with the same opinion, the same way of working prevents burnout.

*‘What I sometimes say laughing is that you only have people who feel good at their job simply because you do things together, provide added value together and continuously feel that you are surrounded and supported.’* (General practitioner).

#### Context of the organization

Healthcare providers who work per performance indicate that their timetable is filled with patients, so there is little time for consultation with external parties. All healthcare professionals confirm that the foreseen resources for these multidisciplinary meetings are not sufficient. Healthcare providers who do not work per performance argue that participating at multidisciplinary meetings cause less of a problem.

The compartmentalization of services is also indicated as a barrier.

One central person or organization who organizes and co-ordinates the inter-professional meetings locally, is perceived as an advantage. This person must be accessible for all local healthcare providers and transcend individual healthcare organizations. Healthcare providers also indicate to have an overview of the local health structure, so that one can see who to contact for which health question. This overview also exposes where the gaps are situated.

The absence of a central secure electronic record in which data can be shared selectively with consent of the patient, is perceived as a deficiency. In the case of the patients’ consent to share data, the system must deliver the possibility to the healthcare provider to determine which data are relevant to share with each individual healthcare provider.

*‘What we do is use the inter-professional consultation as a supporting record where the registered nurses, the physiotherapist, the dietician, the podiatrist, and the occupational therapist all work with, this within our healthcare house. What we’re trying to approach is to see this healthcare facility as an organic entity because then you have mutual contacts and that mind-expanding vision, that continual contact, but then you also continually update the records et cetera.’* (General practitioner).

#### Economic and political context

All healthcare professionals indicate that the current financial regulations do not facilitate inter-professional collaboration. A decent compensation for inter-professional meetings should be provided.

A regional coordinator who organizes and coordinates these meetings is a precondition for a sustainable collaboration on regional level.

*‘What are the practical barriers? First at all the patients not knowing the system… If it is a registered nurse that brings it up, when it’s a general practitioner, if it’s a peer, then they’re inclined to follow them. And then you also have the problem of financing.’* (General practitioner).

## Discussion and conclusion

### Overall findings

Our study sheds light on the willingness for and barriers to collaboration of healthcare professionals in primary care for older adults, with particular attention to occupational therapy as an underused profession. There is little inter-professional collaboration among the Belgian healthcare professionals in primary care, despite the willingness of the healthcare professionals to collaborate.

Knowing each other as healthcare provider is a facilitator to build a sustainable partnership. Inter-professional meetings are essential, but do not always have to be planned. Meeting each other on the work field, short informal contacts between healthcare professionals are also important for information transfer in the context of good care.

Respecting each other within the uniqueness of each one’s job is a precondition of a fluid collaboration. It is not only important to know the content of the job of each involved healthcare professional, also knowing the local healthcare professionals is a facilitator for interprofessional collaboration in primary care. It happens that the care provider discovers hidden care needs and must refer to more suitable care providers to meet that specific care need. Knowing the different professions or being introduced to them by someone they trust is a facilitator for the patient to accept that various health professions are involved.

The older adult is sometimes reluctant to do anything about his situation. Using technics of motivational interviewing can improve motivation. An informed patient is a facilitator to achieve improved outcomes. Therefore, working on health literacy is a factor of success.

In Belgium, the quality of primary care for the elderly can be improved by systematically encouraging local healthcare professionals to gain a better understanding of different care professions. The recently established regional primary care areas may provide support in this regard. A regional primary care area is a network of primary care providers in a geographically defined area consisting of one or more cities or municipalities.

### Components to be addressed to achieve a sustainable collaboration

In accordance with Grol’s (2004) barriers to and incentives for change at different levels of healthcare, we detected several components that need to be addressed to achieve a sustainable collaboration [25].

The detected components in our study on the level of innovation, the patient, the individual professional and the social context, the organizational contex and the economic and political context are in line with the components found by D’Hanis (2015) and Hansson (2018) [17, 24]. D’Hanis (2015) detected lack of patient empowerment and health promotion, multidisciplinary teamwork, integration and continuity between hospital and primary care, and integration and continuity between healthcare and social welfare as barriers to implement an integrated chronic care model in primary care [17]. Hansson (2018) detected obstacles for collaboration on three levels: societal, organizational, and individual. Regulations and financial instruments, lack of time for health professionals, and insufficient communication can hinder collaboration [24].

Wagner’s CCM aims to transform the daily care of patients with chronic illnesses from acute and reactive to proactive, evidence-based, patient centered and planned care. CCM describes the components which interact to promote high quality care for patients with chronic disease [11, 12]. Our study focused on productive interactions, more specifically on communication and collaboration. Regarding the patient, we found that the initial communication about the demand for care of the older adult did not always correspond to the need for care. A recent study on provider discussion on health goals and psychosocial needs shows that older adults report fewer psychosocial issues and health goals, what can lead to missed opportunities [25]. This emphasize that health professionals in primary care who work patient centered must be sensitive for the underlying and non-expressed needs to deliver the correct care and to refer to other healthcare professionals where desirable.

The results of our study are in accordance with the findings of D’Hanis (2015) regarding the fact that the health system and delivery system in primary care in Belgium is still fragmented. This fragmentation results in services that are not yet properly coordinated [17]. Recently, primary care zones have been established in the Dutch speaking part of Belgium. A primary care zone is a geographically defined area, formed by one or more municipalities and managed by a care council. The target of these primary care zones is to establish an effective and high-quality primary care through better coordination [34].

The CCM states that a clinical information system should facilitate and promote the exchange of information between providers and patients [11, 12]. In our study, the healthcare professionals indicate to be willing to share electronic data with other involved professions when explicitly permitted by the patient. This is only on condition that sharing the data improves the quality of the care, and that this happens in a safe manner. The availability and the usage of HICT varies strongly between the various profession groups. General practitioners have the most facilities to register, process and share data in a safe and efficient way.

In the Dutch speaking part of Belgium, the digital BelRAI instruments are gradually implemented. BelRAI is the Belgian implementation of the InterRAI assessment tools. RAI stands for resident assessment instruments. BelRAI instruments map the care needs of vulnerable people in a structured and standardized way with the aim of pursuing qualitative care planning and achieving quality monitoring [35].

The decision support system must provide evidence-based guidelines that should be embedded into daily practice and should be shared with patients to encourage participation [11, 12]. Where it comes to HICT being used for quality support of the intervention, our study also shows major differences between professional groups. Eliminating this imbalance by delivering access to smart software such as an electronic DSS and Evidence Linker is essential to improve the quality of care of older adults.

Self-management support emphasizes the patient’s role in managing health [11, 12]. Our study shows that healthcare professionals underscore the importance of a well-informed patient. Technics of motivational interviewing, health literacy and education are used to achieve an informed older adult, e.g., occupational therapists use HICT to inform older adults.

Community involves linking and using community resources that support healthcare [11, 12]. Recent studies emphasize the significance of social support for the functional status of frail older adults. Finding ways to maintain social support among older adults may offer a promising approach to delay functional decline. Our research demonstrates that healthcare professionals also recognize the value of social support and endeavor to expand the social network among older adults [36].

### Quality of evidence

Keeping in mind the inclusion criteria of the respondents, maximum variety was pursued in terms of employment status, age, and location of the practice (city, suburb, or rural area). The number of respondents were equally divided between professional groups. Since we used purposive sampling, we selected healthcare professionals with an opinion on the subject.

The Johanna Bridge Institute critical appraisal tool for qualitative research (2020) is used to critical appraise our study [37]. We considered congruity between the philosophical perspective and the research methodology, and between the research methodology and the research objectives, the methods to collect data, the presentation and analysis of the data and the interpretation of results. We included a statement about the researcher’s cultural and theoretical orientation to clarify the role of the researcher in the research process. The researcher did not know the respondents, which limited his influence on the research. We included quotes which ensures the presence of the participants in the report. The conclusions are based on interpretation of the data collected through interviews. So, we can state that methodological quality of our study is of such a degree that bias in its design, conduct and analysis are avoided.

### Implications for clinical practice

This study found that healthcare professionals are open to a better collaboration and data-sharing to improve the quality of their interventions.

There is a general need for more collaboration in the care of the older adult. Given the willingness to collaborate between the various care-professions, there is a need for structure and means that facilitate this collaboration in primary care. There should be a concentrated effort to strive for equality where it concerns HICT, so that all healthcare professions can efficiently base their therapeutic procedures on evidence-based research.

When it comes to the possibilities of electronic data capturing, data processing and sharing, there is a clear discrepancy between the various professional groups. The level of availability and accessibility of scientific evidence to support intervention is dependent on the type of profession. The opportunities for efficient collaboration and for detecting evidence that substantiates the intervention are not equal for the different professions.

### Implications for further research and development

Further development must be performed so that all healthcare professions have access to HICT that underpins their therapy with evidence, and that creates the opportunity to share data in a secure way.

There is also a need for a structure and culture that promotes the collaboration between the healthcare professions.

## Conclusion

Belgian healthcare professionals have shown a willingness to collaborate and exchange information to improve outcome measures, if the privacy of the older person is maintained. However, there are several barriers that need to be addressed. Some barriers can be tackled at the regional level through the initiatives of individual healthcare professionals, while others require structural changes at the policy-making level.

### Electronic supplementary material

Below is the link to the electronic supplementary material.

Supplementary Material 1

Supplementary Material 2

## Data Availability

All the interviews were transcribed into text files. The text files containing the transcripts are kept in locked files, accessible only by the corresponding author. The datasets generated and analyzed during the current study are not publicly available and are not available from the corresponding author on request due to reasons concerning participant privacy and confidentiality.

## Abbreviations

CCM: Chronic Care Model
EBP: Evidence-Based Practice
DSS: Decision Support System
HICT: Health Information and Communication Technology
