# Supplementary material for: The willingness and barriers to collaborate in the care of frail older adults: perspectives of primary care professionals
Source: BMC Geriatr. 2023 Aug 11;23:488. doi: 10.1186/s12877-023-04163-y (PMC10422814; doi:10.1186/s12877-023-04163-y)
Supplement: Supplementary file 1 — Supplementary Material 1 [file 12877_2023_4163_MOESM1_ESM.docx]

**Addendum 1:** Interview healthcare professional in primary care

| **Introduction**  Purpose of the interview and practical information  **Reason of care consultation and referral**  What are the most common needs elderly people with chronic health issues consult you?  For what kind of requests do you refer to colleagues? To whom?  To whom do you refer for help concerning functionality?  Can you explain the task of an occupational therapist in primary healthcare? (If they don’t know, informing them)  What can be advantages of implementing occupational therapy systematically in primary healthcare? Disadvantages?  **Collaboration**  Do you find that it is desirable to collaborate with other professions regarding the target group elderly people with chronic health problems?  What do you see as factors for a successful collaboration? Which criteria should be met for a successful collaboration?  What can be improved regarding collaboration?  **Data sharing**  What kind of information on your patient do you want to receive from other healthcare professionals? What exactly from whom?  What do you think is the most efficient way to get this information?  **Electronic data sharing**  What do you think are the advantages and disadvantages of electronic data sharing?  Can you share electronic data on a safe secure way with other healthcare professionals? To whom and what kind of data do you already share?  In your opinion, what needs to be changed to share electronic data efficiently?  **Closure**  Do you want to add something?  Thanking for participating. |
| --- |
