# Supplementary material for: The willingness and barriers to collaborate in the care of frail older adults: perspectives of primary care professionals
Source: BMC Geriatr. 2023 Aug 11;23:488. doi: 10.1186/s12877-023-04163-y (PMC10422814; doi:10.1186/s12877-023-04163-y)
Supplement: Supplementary file 2 — Supplementary Material 2 [file 12877_2023_4163_MOESM2_ESM.docx]

**Addendum 2: Code tree developed in a dialogue between theory-driven and data-driven qualitative analysis**
